# A Structural Hierarchy Matching Approach for Molecular Similarity/Substructure Searching

**DOI:** 10.3390/molecules20058791

**Published:** 2015-05-15

**Authors:** Shu-Shen Ji, Hong-Ju Dong, Xin-Xin Zhou, Ya-Min Liu, Feng-Xue Zhang, Qi Wang, Xin-An Huang

**Affiliations:** 1 Tropical Medicine Institute, Guangzhou University of Chinese Medicine, Guangzhou 510405, China; E-Mails: shushenjijss@163.com (S.-S.J.); zhangfengxue@gzucm.edu.cn (F.-X.Z.); 2 School of Life Sciences, Jilin University, Changchun 130012, China; E-Mail: hongju_dong@jlu.edu.cn; 3 School of Chinese Pharmacy, Guangzhou University of Chinese Medicine, Guangzhou 510405, China; E-Mail: gzzx@gzucm.edu.cn; 4 The First Affiliated Hospital, Guangzhou University of Chinese Medicine, Guangzhou 510405, China; E-Mail: liuyamin2009@gzucm.edu.cn; 5 Institute of Clinical Pharmacology, Guangzhou University of Chinese Medicine, Guangzhou 510405, China; 6 South China Chinese Medicine Collaborative Innovation Center, Guangzhou University of Chinese Medicine, Guangzhou 510405, China

**Keywords:** similarity searching, substructure searching, structural hierarchies

## Abstract

An approach for molecular similarity/substructure searching based on structural hierarchy matching is proposed. In this approach, small molecules are divided into two categories, acyclic and cyclic forms. The latter are further divided into three structural hierarchies, namely, framework, complicated-, and mono-rings. During searching, the similarity coefficients of a structural query and each retrieved molecule are calculated using the hierarchy of the query as the reference. A total of 13,911 chemicals were involved in this work, from which the minimal cyclic and acyclic substructures are extracted, and further processed into fuzzy structural fingerprints. Subsequently, the fingerprints are used as the searching indices for molecular similarity or substructure searching. The tests show that this approach can give user options to choose between one-substructure and multi-substructure searching with sorted results. Moreover, this algorithm has the potential to be developed for molecular similarity searching and substructure analysis.

## 1. Introduction

Structural fragments are commonly used for structural and similarity searches. These searches are used for identifying molecules that possess the same or similar topological fragments for a given query from a chemical library, and also used to establish the property/activity and structure relationships (SPR or SAR) [1,2,3,4,5,6,7,8]. Fragments are generally generated through an atom tracking method. The directly and indirectly connected atoms in a molecule are tracked through atom-by-atom searching, and these continuously connected atoms and their bonds form the final fragments. This method may generate a certain amount of fragments depending on the developer's intention; thus, a larger molecule may have more fragments [1,6]. Substructures mainly refer to the functional groups or moieties that are closely associated with some properties or activities, and thus they can be directly predefined. The smaller fragments inside the substructures are not considered. The concept of object orientation, a terminology widely used in computer programming, enables the user to focus on the objects themselves [7,8]. If a substructure is assigned with sufficient surrounding chemical environment information, it can be treated as the substructure-object and can be used not only in the SPR, SAR, and other multi-dimensional analyses but also in the 2D or 3D similarity searching. This idea enables the user to have more options to operate or use the molecular substructures.

Defining the substructures and assigning them with surrounding chemical environment information are important to implement this idea. A sorted result is desirable. In this work, we refer to the minimal cyclic and acyclic fragments as substructures, endow each substructure with the information of its localization state, use the fuzzy fingerprints as searching indices to conduct similarity and substructure searching, and use the structural hierarchies of the query as the reference to rank the retrieved molecules. This approach is available online [9].

## 2. Results and Discussion

### 2.1. Substructures and Fuzzy Fingerprints

A total of 19,741 cyclic substructures were derived from 13,911 chemicals. Among these, 3247, 7697, and 8797 substructures belong to the complicated, pure aromatic and mono-alicyclic ring groups, respectively. These complicated rings are further simplified into minimal rings that generate 12,522 mono-alicyclic and 3078 aromatic substructures. Therefore, 10,775 aromatic and 21,319 mono-alicyclic substructures are found. Non-ring molecules, side chains, and linkers are dissected into 131,911 minimal linear units. After the unification of one substructure corresponding to one expression and the treatment of fuzzy matching, 631 cyclic and 269 linear fuzzy fingerprints are generated from these substructures. The information of the fuzzy fingerprint and its surrounding chemical environment (fused or isolated for cyclic substructures, side chain, or linker for acyclic substructures) is stored in the fingerprint table for each molecule.

### 2.2. Similarity Searching

When the query is a molecule, this algorithm executes a multi-point (multi-substructure) search. In this procedure, the fingerprints of the query molecule are compared against those of each molecule in the fingerprint table, and the retrieved molecules are ranked in descending order according to Tanimoto coefficients. Although the chemical environment information is limitedly given for the substructures, this approach offers an acceptable result on the matching precision tests, in which over 76% of the query molecules rank 1st, over 92% rank 3rd, over 98% rank 10th and all the others rank 11th to 42nd among the corresponding retrieved molecules (Table 1). Limiting the fuzzy degree improves the matching precision but narrows the searching range. The adopted equilibrium strategy depends on actual need. The result also shows that using the structural hierarchy of the query molecule as the reference to rank the retrieved molecules is a feasible approach.

**Table 1:** Results of the matching precision tests (using each dataset molecule as the query).

Rank	Hits	Rank	Hits	Rank	Hits	Rank	Hits	Rank	Hits	Rank	Hits
1	10,583	8	55	15	9	22	3	29	1	36	1
2	1773	9	40	16	8	23	2	30	1	37	1
3	622	10	30	17	8	24	2	31	1	38	1
4	312	11	22	18	7	25	4	32	1	39	1
5	165	12	17	19	6	26	2	33	1	40	1
6	111	13	13	20	7	27	3	34	1	41	1
7	73	14	11	21	5	28	4	35	1	42	1

### 2.3. Substructure Searching

This approach conducts the substructure searching in two stages: conducting one-point (one substructure and so forth), two-point, or multi-point searching and then ranking the retrieved molecules using the hierarchy of the query as reference. The first stage is to determine whether the other molecules contain the query substructure(s), as this process only places emphasis on specific substructure(s) and it greatly improves the searching speed. The second stage uses the chemical environment information to calculate the Tanimoto coefficient for each hierarchy of the query. We use some examples to explain the searching process. (1) In one-point searching, the canonical SMILES string of “c1ccccc1” represents the isolated substructure of the benzene ring. When this string is used as the entry, the algorithm screens all molecules containing benzene rings. The molecules possessing fused benzene rings are also searched. The current algorithm excludes the latter, so the molecules with only one isolated benzene ring are ranked at top positions; (2) In two-point searching, the SMILES string of “c1ccccc1C” refers to toluene in the chemistry field. However, this algorithm, which complies with the structure explanation of SMILES, treats this string as two substructures of one isolated benzene ring and one methyl group. Therefore, the molecules with one benzene ring and one methyl group are prioritized; (3) In multi-point searching, the SMILES string of “c1ccccc1Cc1ccccc1” is considered a framework that contains two isolated benzene rings and one linker of methylene. Therefore, the molecules with this framework are ranked at top positions.

**Figure 1:**
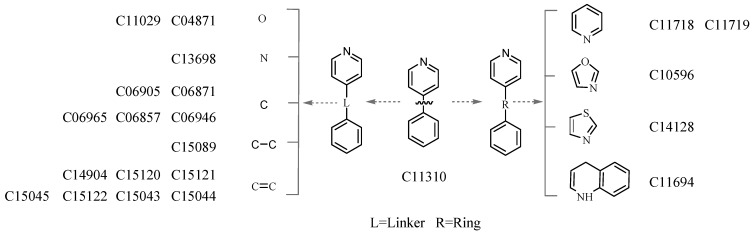
Structural diversity derived from the diverse framework (partial structure of each molecule).

**Figure 2:**
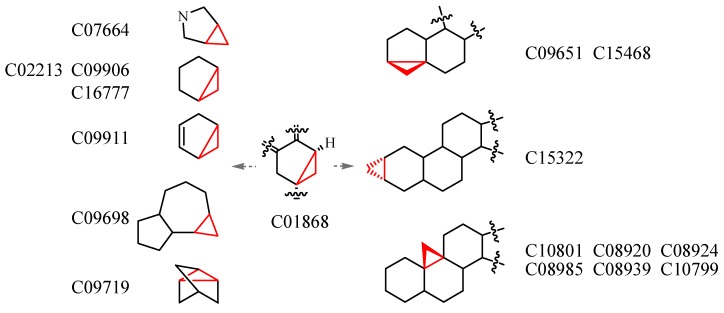
Structural diversity derived from the localization and structure type difference (partial structure of each molecule).

The lack of sufficient connection (or fusion) information between two substructures decreases the matching precision, but it provides a convenient way to investigate the structural diversity of the molecules with the same substructures. For example, the SMILES string of “c1ccccc1c1ccccn1” is structurally considered a framework that contains one isolated pyridine and one benzene ring, and thus the molecules comprising these two rings and a linker or ring(s) are retrieved. Figure 1 indicates that this kind of structural diversity is derived from diverse frameworks, and Figure 2 shows the difference in the fused state of the three-membered ring, in which the query molecule is C01868 that contains a fused three-membered ring in bicycle [3.1.0] hexan. The searching result demonstrates that a three-membered ring can be fused with rings with different sizes (e.g., the six- and seven-membered rings in C15322 and C09698), different types (e.g., azolidine and cyclopentene in C07664 and C09911), and different fusion positions in C15322 and C10801.

## 3. Experimental Section

### 3.1. Chemical Dataset

A total of 13,911 chemicals from the KEGG database (3 September 2010 update) are used as the dataset. These chemicals are classified into drugs, metabolites, and other chemical substances included in KEGG’s biological systems [10,11].

### 3.2. Converting Molecular ConneZction Tables to Canonical SMILES

The canonical SMILES string of each chemical structure is converted from molecular connection table (in MOL format) with the OpenBabel 2.2.2 software [12,13,14,15].

### 3.3. Structure Hierarchies

**Figure 3:**
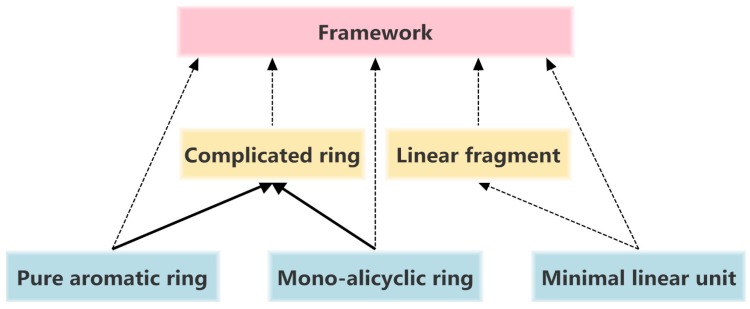
Relationships among the structural hierarchies of molecules with circular substructures. (**⇢** atom connection, **→** bond fusion, 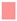 top level, 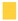 second level, 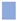 third level).

There are many ways to fragment molecular structures [16]. Based on the connection difference among circular substructures, we classify the molecules with rings into three hierarchies: Framework, complicated (complex) ring, and minimal circular substructure composed of pure aromatic and mono-alicyclic rings. Similarly, the molecules only containing linear substructures are classified into two hierarchies: Linear fragment and minimal linear unit [17,18,19]. Their relationships are illustrated in Figure 3.

The terms used in these hierarchies are interpreted as follows:

*Framework* refers to the skeleton union of the rings and the *Linker*(s). The framework only exists in molecule with circular substructures. One molecule contains one framework at most.

*Complicated ring* is a circular complex characterized by the following properties: (1) contains two rings or more; (2) has one alicyclic ring at least; (3) all component rings are fused or bridged together. This ring has two fundamental forms, namely, fusion occurs in the alicyclic and aromatic rings, in alicyclic rings. Therefore, any complicated ring can be disassembled into smaller rings having one alicyclic ring at least.

*Pure aromatic ring* is any aromatic system in which the aromatic atoms are contiguous.

*Mono-alicyclic ring* is an alicyclic system with only one ring.

*Side chain* is an atom or a cluster of fragments with only one of its terminal ends directly attached to a ring, while a *Linker* is an atom or a chain that connects two isolated rings at both ends.

*Linear fragment*, a union of the same kind of elements, directly comes from the side chain, linker, and any non-ring molecule that does not contain any circular substructure. In its preparation process, any non-carbon element, including saturated heteroatom, metallic element, and halogen element, among others, acts as the separator breaking the whole molecule into several fragments.

*Minimal linear unit* is the maximal collection of the same elements without any branched substructure. Single atom or a single carbon chain is the minimal linear unit. However, a branched fragment is beyond this definition. Given that a branched fragment is made up of only carbon element and possible triple, double, and single bonds, we extract the minimal linear units following the rules generally applied in system nomenclature by the International Union of Pure and Applied Chemistry. Generally, the individual maximum numbers of triple, double, and single bonds are the criteria for determining the main chain in a fragment. After the main chain has been extracted, the remaining substitutes subsequently share the same extraction method until all contained minimal linear units are obtained.

### 3.4. Deriving the Substructures

All rings and line units are treated as substructures. The algorithm for deriving these substructures is described in the following pseudo-code:

		*
SET temp_list substructure_list
READ dataset
FOR each_molecule IN dataset
	DETERMINE molecule_hierarchy
	IF	Framework	THEN
			PUSH ring linker side_chain TO temp_list
	ELSE IF	Complicated_ring	THEN
			PUSH ring side_chain TO temp_list
	ELSE IF	Unit_ring	THEN
			PUSH ring TO substructure_list
			PUSH side_chain TO temp_list
	ELSE
			PUSH Linear_fragment TO temp_list
	ENDIF
ENDFOR
FOR each_ring IN temp_list
	DETERMINE Unit_ring
	IF	TRUE	THEN
			PUSH TO substructure_list	
	ELSE
			GET Unit_ring
			PUSH Unit_ring TO substructure_list
	ENDIF
ENDFOR
FOR each_non-ring IN temp_list
	DETERMINE Unit_line
	IF	TRUE	THEN
			PUSH TO substructure_list	
	ELSE
			REPEAT
				GET longer_linear_fragment
			UNTIL Unit_line
			PUSH Line TO substructure_list
	ENDIF
ENDFOR*


### 3.5. Substructure and Its Chemical Environment

A cyclic substructure can be an isolated state or a fused state in a molecule. Similarly, a linear substructure can act as a side chain or a linker. This kind of chemical environment difference is assigned to the corresponding substructure during the substructure-deriving process.

### 3.6. Fuzzy Fingerprints

We use the symbol of contained element and its total number to encode each substructure. However, one fingerprint mapping may occur for several graphic substructures, and thus we call it fuzzy fingerprint (listed in supplementary file).

### 3.7. Ranking of the Retrieved Molecules

The Tanimoto coefficient is calculated for each hierarchy. The algorithm for ranking the retrieved molecules is described in the following pseudo-code:

		*
READ query_molecule_hierarchy
IF	Complicated_ring	THEN
	RANK Complicated_ring AS first_level
	RANK side_chain AS second_level_or_third_level
ELSE
	RANK	Framework AS first_level
RANK side_chain AS second_level_or_third_level
ENDIF*


### 3.8. Testing the Matching Precision in Similarity Searching

With each molecule in the dataset serving as the query molecule, similar molecules are screened from the dataset, and the rank position of query molecule in the retrieved molecules is counted separately.

## 4. Conclusions

In this work, we propose an approach for similarity/substructure searching and implement it on Linux systems. The test results demonstrate that this algorithm combines the advantages of similarity and substructure searching, especially for substructure searching. It can perform one-point to multi-point searching with acceptable results in chemical big data process [20]. However, the insufficiency of the surrounding chemical environment information limits its matching precision. To successfully apply this algorithm to online analysis, more work is needed, such as the following: (1) endowing the surrounding chemical environment with more connection information between substructures; (2) assigning substructures with hydrophobic, hydrophilic, and electrostatic features; and (3) operating the substructure in a polar coordinate space or a Cartesian space.

## Supplementary Materials

The fuzzy fingerprints of the dataset are provided in the Supplementary Material.

Supplementary materials can be accessed at: http://www.mdpi.com/1420-3049/20/05/8791/s1.
