# Supplementary material for: A Structural Hierarchy Matching Approach for Molecular Similarity/Substructure Searching
Source: Molecules. 2015 May 15;20(5):8791–9. doi: 10.3390/molecules20058791 (PMC6272706; doi:10.3390/molecules20058791)
Supplement: Supplementary file 1 [file molecules-20-08791-s001.pdf]

Supplementary Materials

The Circular Fuzzy Fingerprints Used:

c5n4	c5n1	c4n2	c10n4	c4n1
c3n1s1	c8n1	c6	c3n2	c7n2
c12n2	c12n1s1	c9o1	c6n4	c9n1
c11o2	c10	c4o1	c11n3	c13o1
c6n3	c11n2	c13n1	c7	c14
c7n1s1	c8n2	c15o1n2	c12o1n1	c4s1
c5o1	c12o2	c3n3	c16	c3o1n1
c17n1	c18	c8s1	c12s1	c2n2s1
c2n3	c8o1	c12n1	c1n4	c11n4
c6n2s1	c7o1n1	c12n3	c20	c26
c12o1	c2o1n2	c8o1n1	c15o2	c16o2
c14n2	c15n2	c18o2	c16n3	c10n1s1
c11	c28o4	c12	c11o1n1	c15o1n1
c14o2	c7n3	c4n5	c10n3	c9n3
c18n3	c5n2s1	c13s1	c8o1s1	c7n1
c17	c6o1n2	c22	c9n2s2	c16o1n1
c14n1	c5n5	c15n1	c20n2	O1R1L1
O4L4	O3P3	O1N1R1L1	O1L1	O1L2
O2R1[Fe]	O2R1L1	O2R2	O2L1	O2L2
O7L7	O6L6	O2[Mg]	N1R1L1	N1R1L1=1
N1L1	N2R1L1	N1S1R2	N1R2L1	C4O1
C5N1=2	C5O1	C4N1=2	C2R2L1=2[Fe]	C2L2=2
C4O1=1	C2N2R1	C6=2	C2R2	C3N2
C2S1L1	C6	C2N1L1	C4N1	C4L1=1
C4L1	C3L1	C4N1=1	C5N1L3=3	C2L1
C3S2	C4N2=1	C2N2L1=2	C5	C4N2
C5N1S2	C2L2	C6=1	C3N1S1	C5N1
C2N2R1=1	C3O2	C2N1R1[N+]	C2N1L1=1	C5N1=1
C2R1L1	C5O1=1	C3O1R1	C3O2P1	C2O1L1
C2O1	C15O1=2	C13N5S2	C3N1R1L1=2[Fe]	C3L2=2
C1O2R1	C3O1L1	C4R1	C4R1=1	C1O1L1
C1L2	C4N1S1=1	C3N1	C5=1	C2O1R1
C4R4	C1N1R2	C4S2	C4L1=2	C3
C2O2P1	C1O1R1L1	C15O1L2=1	C1L1	C5O2
C12L1=3	C3O1L1=1	C1O1R2	C3R1	C3L1=1
C3R1=1	C11N5	C4O2	C5L1	C2N1S1R1=1
C2N1R1	C3N1L1	C4	C3N2R1=1	C2O2L1
C13O1N5	C3O1R1=1	C12O1N4L2	C1R1L2=2[Mg]	C2L2=1
C2L1=1	C15O1N1L1=3	C3R1L1=1	C6O1	C1R2
C13O1	C4O1L1	C4O1L1=1	C12O1L1	C4N2=2
C5O6P3L2	C3N1R1	C5N1R2	C2R1L1=2	C4R2L2=3
C3O1	C6N1R2L1=4	C6N1R2L1=5	C4N1[N+]	C3N1S1=1
C2R1	C7L3=3	C6N1	C3R1[N+]	C5L1=1
C3S1L1=1	C17N5S2	C2R2L1=2[Zn]	C4N1L1=3	C2R2L1=2[Mg]
C3N2=1	C4N1S1	C12O1	C7	C12N2

C2L1=1[N+]	C3O2L1	C2O1N1R1	C22N11	C4R2L2=4
C12O2	C10O1L3=1	C1O2L1	C5N1R1	C4N1L1=2
C3O1N1=1	C18N7	C5O1=2	C4R1=2	C2N2R1=2
C5N1L3=4	C2N1L2=2	C4N1L3=2	C6N1R1L2=4	C3R1L3=2
C5N1R1L2=4	C9O3	C4S1	C1N2L1	C3L4=2
C3L4=3	C3N1L1=1	C3L2=1	C3O1N1	C3R1=1[N+]
C3L1=2	C7O2L1	C4=1[N+]	C2O3P2	C1O2N1[Fe]
C1O2N1L1	C9O1N2L1	C5N1L1	C7N4L1	C5O2[Fe]
C4O2L1	C6O2N2R1L1	C4O1N1L1	C5N1L3=1	C6N1R2L1=3
C5N1R1L2=1	C5N1L3=2	C5N2	C3O2R3L1	C3N1R1=1
C1N1R1L1	C1N1L1	C37O1=6	C32O1L1=7	C11O1L1
C2N1	C24O6N6	C5N2R2	C3O1N1R2L1	C5O1N2R1L1
C4=1	C16O4L4	C10O1N3L1	C1O1L2	C2N1R2=1
C3R2	C4[N+]	C13O1N1	C4N1R1	C1R1L1
C4N1L1	C15N6S2	C2S2	C2N1R2	C1N2R2=1
C3R2=1	C1N2S1R1=1	C3N1S1R1	C3S1R1	C2O2R1
C2O1R2	C8	C7N1	C3O1N1P1	C3O1S1
C1O1N1R2=1	C4O1N1	C4[S+]	C4R1L1	C4O1N1=1
C2N2L1=1	C2R1L1=1	C3L2	C1N1S1R2=1	C5[N+]
C9O2N3[Fe]	C1R1	C6L2=1	C1N2S1R1	C2N1S1R1
C3O3	C1O2R1[Bi]	C23O1L2=4	C2O2R2L2	C3N1[N-]
C1S1R2	C9O1L2=3	C2S2[As]	C11O1N3L2	C17O1N2R1=3
C18O1L2=5	C3=1	C2S5	C1N1L2	C11N4S1R1
C6N3L2	C8N2S1R1L1	C8N4	C7O1N2R1	C7O1N2L1
C4S2=2	C7L1	C9O1=1	C2R1[N+]	C3N1=2[N+]
C2O1L1=1	C6N1=1	C15O2	C6L1	C8N1L1
C3O1L2	C1O1R1L1=1	C2O1L2	C2L3	C6O6L5
C3R1L1	C1L1=1	C4O1R1=1	C10	C14=3
C4L2=1	C1L3	C2L1[N+]	C2N1L2	C6L1=1
C12O3=1	C6O1=1	C4L2	C7L1=1	C8L1=2
C10L1	C7L2=1	C8N1R1=1	C1N1L1=1	C7N1R1
C10O1L1	C9O1L1=1	C7=1	C17O1L1=1	C3N1R1L1
C6N1R1	C8N1=1	C3O1=1	C8N1R1	C5=2
C2N1R1L1	C8L1=1	C2O1R2=1	C5L2=1	C3L3
C4L3=1	C8L1	C5O1L1	C10=2	C5R1L1=1
C6L2=2	C6L2	C4N1R2	C4L3	C3R2[N+]
C5L1=2	C3O1R3L1	C3R2L1	C2O1R2L1	C11=3
C12O3L1=2	C11N1=2	C6R1=1	C8O1R1=2	C8=1
C6O1L1	C4O1R1L1	C14O2	C3N1L1=2	C2O1R1L1
C11O1R1=1	C9O2R1L1	C7O1N2R1=1	C6O1N1R1L1=1	C9N3R1=1
C5O1N2R1L1=1	C4N1R1=1	C16O2L2	C5O2R2	C4O2R2
C5O3R2	C6O2R2	C3O2L2	C3O2R2L1	C5O2L1
C4O2R2L1	C1O1R1	C1R1L2	C4O2R4	C10O2=1
C7N1=1	C6O2L1	C6O2L2	C4O2L2	C9O3L1
C10O2	C3O2=1	C6R1	C10N3	C13O1N3R2=2
C3O1R2L1=1	C2S2L1	C6N2	C13N3L1=2	C6R2L1
C10N1R1L2=2	C13N4	C9N2L1	C4O1N1R1	C3N1S2

C2R2L1	C5R1=1	C1N1R1L1=1	C4R2	C5R1
C3N2S1	C5S1	C3S5	C4O1S1=1	C9O1L1
C2O2S1L1	C4S1=1	C4S2=1	C18N8L2	C15N1L1=3
C2S1R1	C2R1L2=3[Mg]	C6O1N1	C16O1N1R1L1=3	C12O1L1=1
C22O1L1=3	C7O2	C2O2L2	C2O2[Sb]	C5N1S1
C5O2R1L1	C7L1=1#2	C6L1=1#2	C3O3P2	C12=2#2
C8L1=1#2	C3S3	C3N1R1L1=2[Co]	C3N1R1L1=1[Co]	C4N1L4=3
C3L4	C9N4L1	C12O3N3	C16N7	C12O1N4L1
C2N1R1=1	C3N3=2	C5O1L1=1	C1R1L2=2[Zn]	C11O1
C14=4	C10O1L2=3	C5O1L2=1	C11O1L2=3	C12O1L1=3
C13O1L1=3	C11O1=1	C13O1=1	C33O1N15	C21O1N9
C2O1N1R2L1	C16N7L1	C15O1=1	C19N9L1	C16O1N6R1L1
C17O1N7	C5O1N2R2	C2O1N1R1L2	C8N4R5	C9O1N1S1R2
C5N3R4L1	C11O1N5R1L2	C7N4R2L2	C12N6R6	C7O1N1S1R1
C8N4R6	C21O1=7	C17N1L1=4	C13O1L1	C32O1L1=6
C17N1L1=3	C15N1R1=3	C19O1N8	C1S1R1[N-]	C1O1S1R1
C2O2[Pt]	C2O3P2R1L2	C27O1=5	C5R1L2	C3O1N1L1
C2N2L1	C4N2R4	C1R3	C68O4	C2N5R2L1=5
C7O1	C8O6R2	C2S1L1=1	C1S1R1	C13N1L2=3
C11O1R1	C2S1R2	C2[S+]	C17=1	C3N2R1=2
C3N2R1	C4L1=3	C1S2L1	C4R3L1=1	C2R2L1=3[Fe]
C7N3L1	C13N1R1L1=3	C13O1L1=2	C15O1=3	C25O1N11
C17O1=2	C19N1=5	C11O1N4L1	C6N2R2	C17O1N5L2
C10N2=2	C14N5	C11O1N4	C12N4	C4N2R1
C12O1N3S2	C8O1N3L1	C18O2N6	C13N6L1=1	C12N3S3
C8N3S1L1	C27O1R1=9	C8N4R2L2	C6N3L3	C43O1=8
C13O1=2	C26O1N1=2	C13L1=4	C17O1=4	C11O1N3S2L1
C7O1N3L2	C16O2N6L2	C22O1L1=5	C13N4L2	C4N2L1=1[Co]
C2L3=1	C3N2L1=2[Co]	C2O2[B-]	C2O4P2L2	C2R1L1=2[Mg]
C2R1L2=2	C20N6S2	C18N5S2	C6O1L1=1	C1L2=1
C5L2	C8O1N3	C6L3	C11=2	C16O2N3L1
C17O1=1	C10=1	C7=2	C8R1=2	C6R1L1=1
C4O1N1S1	C8R1=1	C4O2P1L2	R1L1	R1L2
R2L1	R7L1	R1L1[C-]	L2	L3
L2[C-]				

The Linear Fuzzy Fingerprints Used:

C1	C2	C4	C3	C5
C2=1	C6	C3=1	C20=4	C4=1
C16	C7	C18=1	C1N1#1	C8=2
C44=11	C16=4	C9=4	C18	C5=1
C8=1	C7=1	C12=3	C1=1	C8
C5=2	C6=2	C24=6	C9=5	C3#1
C14=4	C9	C21=5	C10=1	C12#2
C16=1	C21=4	C2#1	C2=2	C10
C18=2	C17	C12	C11	C6=1

C15	C36=9	C20	C18=9	C13
C4#1	C14	C15=4	C12=1	C40=10
C18=3	C22	C20=3	C13=3	C11=3
C9=2	C32=8	C7=2	C11=1	C28=7
C20=5	C31=1	C4=2	C11=2	C10=2
C14=1	C12=2	C32=9	C32=10	C32=11
C32=12	C32=13	C25=10	C25=11	C17=1
C35=11	C22=6	C8=3	C17=8	C11=5
C19=4	C9=1	C18=1#1	C5=1#1	C6#1
C7=1#1	C22=1	C24	C24=1	C31
C26	C29	C28	C30	C33
C13=3#2	C14=2#2	C4=1#1	C6#2	C17=3#2
C10=1#3	C13=1#4	C17=2#2	C7=1#2	C10=1#2
C16=3#1	C6=1#2	C7#3	C13=2#3	C18#1
C5#2	C6#3	C13=1#5	C30=2	C15=7
C20=9	C19=9	C16=7	C18=10	C16=8
C21=10	C25=12	C10=3	C13=2	C16=3
C12=4	C10=4	C7=3	C15=1	C15=2
C16=5	C17=5	C18=6	C17=6	C16=6
C19	C21	C9=3	C9=1#3	C17=2
C22=4	C17=3	C76=2	C13=7	C13=6
C14=3	C22#1	C3=2	C17=7	C18=8
C28=2	C29=10	C13=1	C24=10	C32=14
C23=11	C24=7	C18=4	C22=5	C24=5
C24=4	C20=2	C28=13	C20=1	C22=2
C22=3	C8=4	C41	C17=1#2	C16=2
C17=4	C9=2#2	C23=2#5	C23	O1
O1=1	N1	N1=1	N1#1[C-]	N2#1
N1#1[N+]	N1#1	P1	S1	S1=1
=1[NH-]	=1[N+]	=1	=1[Se]	=1[NH+]
=1[As]	#1[C-][OH+]	#1[NH-][N+]	#1[C-][N+]	Cl
F	Br	I	[OH]	[O-]
[S+]	[Fe]	[Mn]	[Zn]	[Cu]
[Ca]	[H+]	[N+]	[Mo]	[Co]
[Cl]	[KH+]	[H]	[Ni]	[Mg]
[SH-]	[*H]	[N]	[Hg]	[I-]
[F-]	[W]	[O]	[Br]	[OH-]
[Na]	[NH+]	[Cd]	[As]	[Se]
[NH]	[Te]	[S]	[Pb]	[Ag]
[Pt]	[Si]	[Li]	[K]	[I]
[N-]	[Au]	[Sn]	[P+]	[V-]
[B]	[Ge]	[Bi]	[Al]	[Gd]
[Xe]	[C-]	[Rb]	[B+]	[B-]
[Ba]	[Sr]	[Ru]	[Tl]	[Cr]
[Sb]	[Rn]	[Ra]	[Be]	
